# A Review of Polymer-Based Environment-Induced Nanogenerators: Power Generation Performance and Polymer Material Manipulations

**DOI:** 10.3390/polym16040555

**Published:** 2024-02-18

**Authors:** Shuanghong Xie, Huping Yan, Ronghui Qi

**Affiliations:** 1 Key Laboratory of Enhanced Heat Transfer and Energy Conservation of Education Ministry, School of Chemistry and Chemical Engineering, South China University of Technology, Guangzhou 510640, Chinayanpingrongjiang@163.com (H.Y.); 2 Guangdong Provincial Key Laboratory of Fuel Cell Technology, School of Chemistry and Chemical Engineering, South China University of Technology, Guangzhou 510640, China

**Keywords:** environment-induced nanogenerator, polymer-based, power generation performance, ambient energy harvesting, ionic hydrogels

## Abstract

Natural environment hosts a considerable amount of accessible energy, comprising mechanical, thermal, and chemical potentials. Environment-induced nanogenerators are nanomaterial-based electronic chips that capture environmental energy and convert it into electricity in an environmentally friendly way. Polymers, characterized by their superior flexibility, lightweight, and ease of processing, are considered viable materials. In this paper, a thorough review and comparison of various polymer-based nanogenerators were provided, focusing on their power generation principles, key materials, power density and stability, and performance modulation methods. The latest developed nanogenerators mainly include triboelectric nanogenerators (TriboENG), piezoelectric nanogenerators (PENG), thermoelectric nanogenerators (ThermoENG), osmotic power nanogenerator (OPNG), and moist-electric generators (MENG). Potential practical applications of polymer-based nanogenerator were also summarized. The review found that polymer nanogenerators can harness a variety of energy sources, with the basic power generation mechanism centered on displacement/conduction currents induced by dipole/ion polarization, due to the non-uniform distribution of physical fields within the polymers. The performance enhancement should mainly start from strengthening the ion mobility and positive/negative ion separation in polymer materials. The development of ionic hydrogel and hydrogel matrix composites is promising for future nanogenerators and can also enable multi-energy collaborative power generation. In addition, enhancing the uneven distribution of temperature, concentration, and pressure induced by surrounding environment within polymer materials can also effectively improve output performance. Finally, the challenges faced by polymer-based nanogenerators and directions for future development were prospected.

## 1. Introduction

Fossil fuels such as petroleum, natural gas, and coal are non-renewable resources. Their prices have risen sharply in recent years, and pollutants such as CO_2_, SO_2_, SO_3_, and volatile organic compounds (VOCs) released during fossil power generation have intensified the greenhouse effect, ozone layer depletion, acid rain, and environmental pollutions [1,2]. It is imperative to reduce dependence on fossil fuel and explore innovative green and renewable energy sources [3,4]. Notably, our surrounding nature holds an extensive reservoir of green and clean energy, encompassing mechanical potential from friction and vibration, thermal potential from evaporation, and chemical potential from salinity or concentration gradient, etc. Utilizing these energy sources for electricity generation is considered a potential solution to address the escalating energy crisis [5]. Nanodevices made from nanomaterials offer a pathway for achieving efficient energy collection, storage, and utilization on a small scale [6]. They boast advantages such as a straightforward structure, diminutive size, and high-power density [7,8]. The basic concept of environmental nanogenerators is to capture green ambient energy in human life and convert it into internal polarization within power-generating materials. When the power-generating material is connected to an external electrical appliance, the potential difference generated by the polarization of the material drives the flow of free electrons, thereby outputting observable voltage and current.

**Figure 1:**
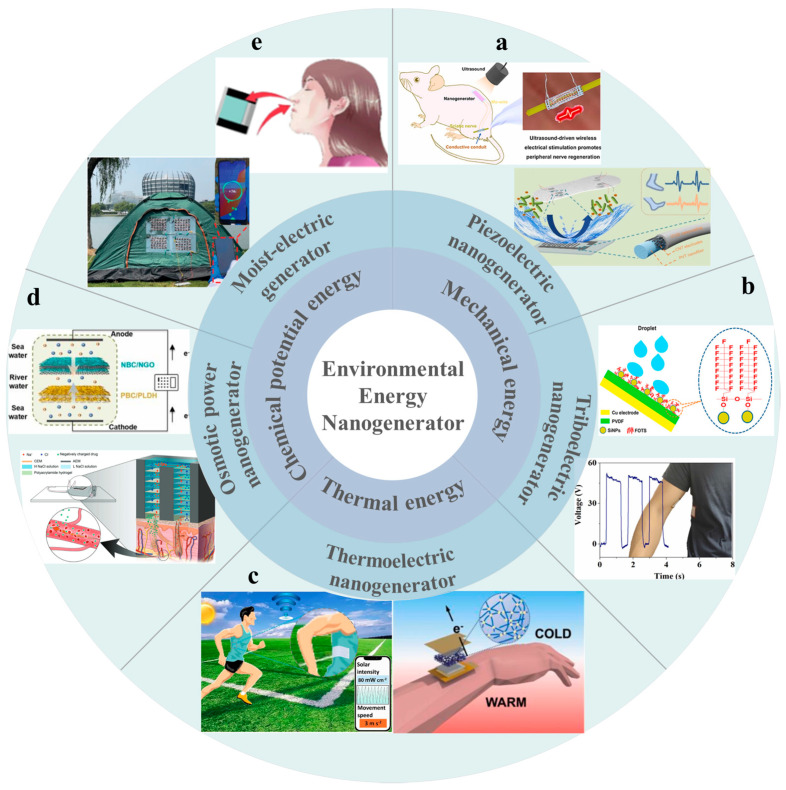
Different nanogenerators and their applications: using mechanical energy: (**a**) PENG: for the manufacture of implantable medical devices [25] and human gait monitoring sensors [26], (**b**) TriboENG: for the collection of friction energy between droplets and triboelectric materials [27] or between different triboelectric materials [28]. (**c**) ThermoENG using thermal energy: it is used to manufacture self-powered sensors [29] and wearable devices [30]. Using chemical potential energy: (**d**) OPNG: used to collect energy between different concentrations of liquids [31] and promote in vitro drug delivery [32], (**e**) MENG: used to collect human respiration [33] and humidity energy in the environment to charge smartphones [34].

Polymers, high-molecular-weight substances resulting from the cross-linking polymerization of numerous monomer molecules, exhibit outstanding flexibility, durability, and biocompatibility. Their ease of processing and molding renders them advantageous in the material selection for nanogenerators [9]. Application fields of polymer nanogenerators encompass battery-free Internet of Things devices [10,11], wearable devices [12,13], implantable devices [14,15], and sensors [16,17]. As shown in Figure 1, recent developments in nanogenerators employing polymers as power generation materials including triboelectric nanogenerators (TriboENG), piezoelectric nanogenerators (PENG), thermoelectric nanogenerators (ThermoENG), osmotic power nanogenerator (OPNG), and moist-electric nanogenerators (MENG). Specifically, the TriboENG captures friction energy generated during vibration through the triboelectric effect between different materials, achieving external energy output via the electrostatic effect [18,19]. The piezoelectric nanogenerator transforms pressure changes in the vibration process into ion or dipole polarization within the material through the material’s piezoelectric effect, ultimately obtaining a corresponding potential difference at both ends of the material [20,21]. Thermoelectric generators convert environmental temperature differences into electrical energy through the Seebeck effect (materials with electrons and holes as carriers) [22] and the Soret effect (materials with ions as carriers) [23]. OPNG utilizes an ion-selective permeation membrane to separate solutions of different concentrations, converting the chemical potential energy difference into distinct ion polarization phenomena on both sides of the film. Ultimately, electric energy is output through the redox reaction at the electrode. MENG collects environmental humidity energy, converting it into ion polarization within the material through the asymmetric moisture absorption/evaporation of the power generation material, and subsequently outputting current [24].

Until now, there are some published review papers about polymer-based nanogenerators [35,36], which explored the performance improvement methods [37,38] and application prospects of different materials [39] in nanopower generation systems. However, these reviews merely regarded PENG and TriboENG into nanogenerators. Therefore, there has been a lack of the complete type of nanogenerator systems (including PENG and TriboENG, ThermoENG, OPNG, and MENG). In this paper, we divided the above types of power generation systems into nanogenerators by analyzing their generator mechanisms. According to the sources, we divided polymers into two types: synthetic polymers (SP) and natural polymers (NP). Due to different energy storage modes of SP, we further divided them into three categories: dielectric polymers, conductive polymers, and ionic hydrogels, which are the focus of this article. We further explored the application of these polymers in different types of nanogenerators and performance enhancement methods.

This paper provides a systematic review of polymer-based nanogenerators used in piezoelectric, triboelectric, thermoelectric, salinity/concentration or humidity gradient energy harvesting. It delineates the principles of electricity generation, performance characteristics, and the research progress of diverse generators employed in various power generation systems. In Section 2, the electricity generation principles of nanogenerators driven by mechanical, thermal, and salinity gradient energy are summarized. Section 3 explores the key materials and performance indicators across different generator types. By summarizing methods to modulate the performance of various generators, the paper concludes that the coupling of different energy sources can be achieved through the meticulous selection of materials, enhancing the power output density of nanogenerators while ensuring the stability of the power generation system.

## 2. Working Principle of Polymer-Based Nanogenerators Using Environmental Energy

Environmental energy nanogenerators function by employing materials with specific structures or characteristics to convert mechanical energy, temperature differences, or chemical potential energy variations in the environment into a polarization phenomenon of charged particles within the material. This polarization is then utilized through a device to produce an external output, ultimately accomplishing the conversion of environmental energy into electrical energy [40]. According to Wang’s expansion of Maxwell’s displacement current equation in 2017, the nanogenerator’s current comprises both displacement current and conduction current, as expressed in Equation (1). The conduction current results from the flow of conductor electrons [41], while the displacement current $I_{d}$ arises from changes in the environmental conditions of the power generation material, encompassing the external electric field action term and the polarization field term, as presented in Equation (2):

(1) $$I_{t}=I_{c}+I_{d}=I_{c}+\int_{S}\;\left(\frac{\partial D}{\partial t}\right)\cdot dS$$

(2) $$\frac{\partial D}{\partial t}=t\frac{\partial E}{\partial t}+\frac{\partial P_{s}}{\partial t}$$

where $I_{t},\;I_{c},\;I_{d}$ represent the total current, conduction current, and displacement current, respectively. $D,\;E,\;P_{s}$ are the displacement field, electric field and polarization field caused by surface electrostatic charge, respectively. The partial derivative $\frac{\partial P_{s}}{\partial t}$ is directly related to the output current of the nanogenerator.

### 2.1. Piezoelectric Nanogenerator

**Figure 2:**
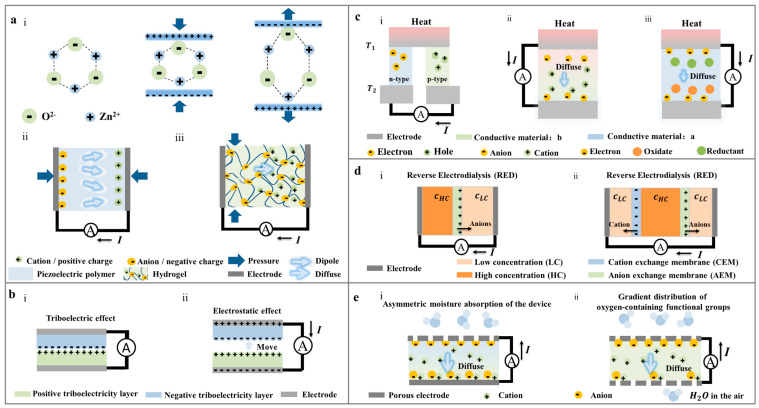
Power generation principles of different nanogenerators: (**a**) The power generation principle of PENG with different types of materials: (i) inorganic piezoelectric crystals (ZnO) with asymmetric charge centers [42], (ii) Piezoelectric polymer with permanent dipole moment [43], (iii) Ionic hydrogel [44]. (**b**) The power generation principle of TriboENG [45]: (i) Triboelectric effect, (ii) Electrostatic effect. (**c**) Different types of ThermoENG [46]: (i) Conductive polymer-based thermal diffusion generator, (ii) Ion hydrogel-based thermal diffusion generator, (iii) Ionic hydrogel-based thermocouple generator. (**d**) The structure of OPNG [47]: (i) only one selective cation permeable membrane and (ii) both anion and cation selective permeable membranes: positive and negative ions move from high concentration side to low concentration side through cation selective permeable membrane and anion selective permeable membrane, respectively. (**e**) The formation method of MENG: (i) the asymmetric distribution of hygroscopic materials [48], (ii) the gradient distribution of oxygen-containing functional groups.

The piezoelectric effect was initially observed in inorganic crystal materials possessing asymmetric charge centers. The asymmetrical displacement of positive and negative current carriers during compression results in material polarization. The piezoelectric effect is categorized into positive piezoelectric effect and inverse piezoelectric effect, as depicted in Figure 2a. When an external force is applied, the piezoelectric material exhibits a measurable potential difference due to the material’s internal deformation, referred to as the positive piezoelectric effect. The potential generated by the positive piezoelectric effect is contingent upon the force direction applied to the material. Consequently, alterations in force direction lead to changes in the polarization phenomenon within the material, resulting in corresponding variations in the piezoelectric potential.

Many organic piezoelectric polymers and ionic hydrogel materials exhibit piezoelectric effects. For organic piezoelectric polymer materials, the prerequisite for the piezoelectric effect is the presence of a permanent dipole inside the material [49]. Under stress, changing the distance/direction between atoms or molecules in the polymer alters the electric dipole moment, subsequently modifying the polarization intensity and direction of the crystal. This process realizes the conversion between mechanical energy and electrical energy [35]. In ionic hydrogel materials, negatively charged groups polymerize to form a hydrogel skeleton structure, characterized by smaller ion mobility. Conversely, positively charged cationic groups, with a smaller size, exhibit larger ion mobility. Consequently, under external pressure force [44], an overall potential difference appears in the piezoelectric hydrogel material. The piezoelectric polymers commonly investigated in research can be categorized based on the material’s conductivity into two types: organic ferroelectric polymer materials with insulating properties and ionic hydrogel materials with conductive properties [44]. The piezoelectric properties are closely linked to the intensity of the piezoelectric effect in these materials under the influence of a force field or electric field, often expressed by piezoelectric constants $d_{ij}$, where *i* refers to the direction of the electric field, and *j* refers to the direction of stress or strain. When the subscript of the piezoelectric coefficient is “33”, it indicates that the polarization direction is the same as the direction of force applied during measurement, both perpendicular to the horizontal plane [35]:

(3) $$d_{33}=\frac{\Delta S_{3}}{\Delta E}$$

where $\Delta S_{3}$ represents the thickness strain of the material, and ΔE represents the magnitude of the applied/generated electric field. Generally, the unit of ${|d}_{33}|$ is “C/N” or “m/V” [35], and $d_{33}>0$ for inorganic materials and $d_{33}<0$ for piezoelectric polymer materials.

### 2.2. Triboelectric Nanogenerator

The operation of a triboelectric nanogenerator primarily involves the triboelectric effect and electrostatic effect. When two materials with different triboelectric series (friction layers) come into contact and rub against each other, as shown in Figure 2b, charged particles transfer from one material surface to the other, resulting in positive and negative charges on the two friction layers, respectively. This leads to the generation of an internal capacitance “C” with a charge of “Q” between the friction layers of the two materials. This phenomenon is known as the triboelectric effect or contact electrification (CE). When the two friction layers are in contact, the spacing between the positive and negative electrostatic charges generated by the friction can be neglected. The net charge due to friction on the surface of the friction layer with dielectric properties tends to persist for an extended period. Under the influence of electrostatic attraction, an induced charge appears on the back electrode connected to the friction layer to balance the internal electric field.

(4) U=QC=Q(εrS4πkd)

where $\varepsilon_{r}$ represents the relative dielectric constant of the dielectric, *S* denotes the positive area, *k* is the electrostatic force constant, *d* represents the distance between the two plates, and *Q* signifies the amount of charge carried by the capacitor.

When dielectric polymers are used as triboelectric materials, the electrical disparity between the triboelectric materials directly establishes the upper limit of triboelectric property output. Wang et al. conducted systematic tests using copper and mercury as friction contact materials in a vertical contact friction mode, evaluating commonly studied polymer materials [50] and inorganic non-metallic materials [45]. They established triboelectric series for materials within corresponding categories. By selecting two materials with significant differences in the triboelectric series as the friction layers, the number of electron transfers in the contact electrification (CE) process can be increased, resulting in improved output performance [51].

### 2.3. Thermoelectric Nanogenerator

A thermoelectric nanogenerator (ThermoENG) is a small device based on the thermoelectric effect and driven by a temperature gradient. It can be classified into two categories, depending on the presence or absence of chemical reactions: thermal diffusion generators and thermocouple generators (Figure 2c). In thermal diffusion generators, carriers can be categorized into electronic thermoelectric (e-TE) with electrons (or holes) as carriers and ionic thermoelectric (i-TE) with anions and cations in solution as carriers, depending on the types of carriers in thermoelectric materials. E-TE typically employs conductive polymers as power generation materials, relying on the first thermoelectric effect—the Seebeck effect. When the two ends of the conductor experience different temperatures, carriers (electrons or holes) at the hot end possess higher kinetic energy. These carriers diffuse from the hot end to the cold end, accumulating at the cold end to create a thermoelectric potential [22,52]. E-TE generators generally use a combination of P-type and N-type semiconductors as power generation materials, as depicted in Figure 2c. The thermoelectric electromotive force ΔU and the temperature difference ΔT at both ends of the conductor exhibit a linear relationship, expressed as [53]:

(5) ΔU=S×ΔT

where S denotes the Seebeck coefficient of the conductor. When the carrier in the material is a hole, the Seebeck coefficient of the material is positive, indicating a P-type conductor. Conversely, when the carriers in the material are electrons, the Seebeck coefficient is negative, designating an N-type conductor.

Another type of thermal diffusion generator, i-TE, typically utilizes ionic hydrogel as the power generation material, relying on the Soret effect as the internal power generation principle. The Soret effect takes advantage of the different radii of anions and cations. Ions with smaller radii encounter less movement resistance during migration from the hot end to the cold end under a temperature gradient, resulting in greater mobility. Consequently, due to the disparate mobility of anions and cations, ions with higher mobility accumulate at the cold end, while those with lower mobility accumulate at the hot end. This leads to the phenomenon of ion polarization, converting the internal energy between different temperatures in the environment into electrical energy for output [23,54].

The thermocouple generator, also known as a pyrogen battery, is illustrated in Figure 2c. Typically, the pyrogen battery consists of positive and negative electrodes and ionic hydrogel materials containing redox couple pairs. While the internal composition of the power generation material is uniform, the two ends are exposed to different temperature environments. There exists a thermoelectric potential difference between the hot side and the cold side. Under the influence of thermopower, carriers (electrons, holes, or ions) within the hydrogel migrate from the hot end to the cold end. Electrons, in particular, move from the oxidation reaction side (cold end) to the reduction reaction side (hot end) through the external circuit, aiming to establish equilibrium within the material [55].

### 2.4. Osmotic Power Nanogenerators Using Salinity Difference

The osmotic power generator based on reverse electrodialysis (RED) can be traced back to 1954 [56], as shown in Figure 2d. Ions with electrical properties opposite to the ion-exchange membrane diffuse from high concentration areas to low concentration areas under the influence of concentration differences. The key material in RED is polymer membrane with a high density of charged ionic functional groups. Based on the electrical properties of the film, it can be categorized as a cation-selective membrane (negatively charged) or an anion-selective membrane (positively charged) [56]. When the ion-selective membrane is in contact with the electrolyte solution, the fixed charge on the membrane surface attracts oppositely charged ions and repels similarly charged ions. This allows countercharge ions in the high concentration region to diffuse through the ion-selective membrane to the low concentration region. The ion polarization phenomenon occurs in the solution on both sides of the membrane, leading to the appearance of the potential difference. In an open circuit, the sum of the membrane potential of each ion-exchange membrane represents the open-circuit voltage of the device. When the circuit is closed, a redox reaction occurs near the electrode and the solution, causing electrons to move directionally between the two electrodes, resulting in a current [57]. The OPNG system based on RED technology can also be coupled with the thermoelectric system. The temperature difference in the solution on both sides of the ion-exchange membrane leads to varying evaporation rates of the solution, enabling the coupling of the thermoelectric and OPNG system for enhanced power generation performance. By integrating photothermal technology, it can be employed for solar energy collection, seawater desalination, and more [58,59]. The ion transport flux in the RED battery can be expressed by the extended Nernst–Planck equation [60]:

(6) $$J_{i}=vC_{i}=D_{i}\frac{dC_{i}}{dx}-\frac{z_{i}FC_{i}D_{i}d\varphi }{dx}$$

where v is the convection velocity of the solvent, *x* is the film thickness, $C_{i},\;D_{i},\;z_{i}$ are the concentration, diffusion coefficient, and valence state of ions (*i*), respectively. φ is the potential, and *F* is the Faraday constant. The term $vC_{i}$ represents the flux of convective transport of ions with the solvent under osmotic pressure.

The performance of OPNG is influenced by factors such as membrane surface charge density, permeability, membrane resistance, and thickness. When an electric double layer overlap occurs, the nanochannels inside the power generation material acquire a single charge, transforming them into ion-selective channels. It is generally accepted that a higher surface charge density or smaller pore size enhances ion selectivity, resulting in a larger voltage in reverse electrodialysis (RED). However, the increase in membrane permeability may lead to a decrease in the current of the RED system [61]. Nevertheless, ion selectivity is mainly determined by the interplay between surface conductivity and bulk conductivity. This suggests that smaller pores may exhibit lower ion selectivity and permeation potential compared to larger pores [47,62].

### 2.5. Moist-Electric Nanogenerators

Moist-electric generation technology is an innovative power generation approach involving two primary steps [24,48]: (1) Power generation materials used in MENG absorb water vapor from the air under the influence of humidity differences between its two ends. This results in ion hydrolysis and separation of oxygen-containing functional groups within the material. (2) Facilitated by ion selectivity due to electric double layer overlap [63] and the ion concentration gradient caused by humidity variation, mobile ions (typically H^+^) diffuse from high humidity end/high concentration side to low humidity end/low concentration side, creating an ion polarization phenomenon. This transformation converts the associated Gibbs free energy change into a potential difference during the water diffusion process from the high humidity side to the low humidity side [64]. In conditions of high humidity, the driving force for water evaporation and absorption is stronger, leading to a greater number of protons absorbed by water molecules and dissociated by wet electrical materials. Through thermodynamic analysis of the diffusion-drift balance of MENG, its output voltage ($U_{out}$) could be expressed by Equation (7) [65,66]:

(7) $$U_{out}=\frac{RT}{nF}\ln(\frac{c_{ion,high}}{c_{ion,low}})$$

where *R*, *T*, *n*, and *F* represent the gas constant, temperature, valence of compound, and Faraday’s constant, $c_{ion,high}$ and $c_{ion,low}$ the concentrations of mobile ions on the high concentration side and low concentration side, respectively.

As it necessitates the adsorption and phase change process of water vapor, the power generation materials utilizing ambient humidity should contain abundant oxygen-containing functional groups. In current research, hydrogels such as polypyrrole (PPy), polydopamine (PDA), poly (4-styrenesulfonic acid) (PSSA), polyacrylic acid, and polyvinyl alcohol (PVA) are well investigated and used due to the presence of hydrophilic and hygroscopic oxygen-containing functional groups like hydroxyl (–OH), carboxyl (–COOH), and sulfonic acid group (–SO_3_H), which can dissociate into a significant amount of H^+^ upon absorbing water vapor [67].

Elevating relative humidity enhances both the power generation driving force and the number of dissociated protons, thereby improving the power generation performance of the wet power system. The techniques for creating a humidity gradient can be categorized into two main approaches: asymmetric evaporation of water in homogeneous materials and the asymmetric distribution of hygroscopic materials in heterogeneous materials. In homogeneous materials, the design primarily focuses on the contact area between the power generation material and the surrounding air. The portion with a larger contact area exhibits reduced resistance to moisture absorption/evaporation within the power generation material. As a result, distinct moisture absorption/evaporation rates emerge at the two ends of the power generation material, ultimately giving rise to a humidity gradient. On the other hand, the asymmetric distribution of hygroscopic materials involves the uneven distribution of hydrophilic functional groups within the hygroscopic material itself or its internal structure. This disparity leads to differential adsorption of water vapor from the air by power generation medium.

## 3. Dielectric Polymers for Environment-Induced Power Generation

**Figure 3:**
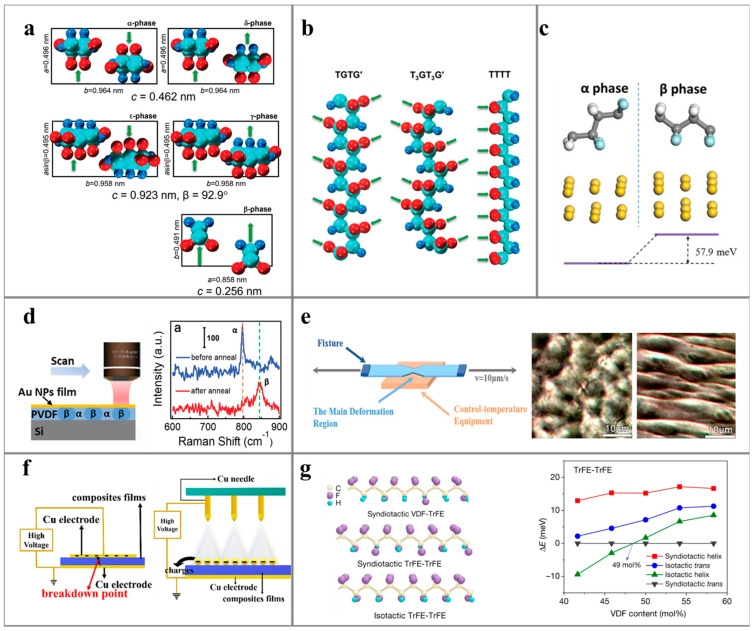
Methods and schematic diagrams for improving the piezoelectric performance of PVDF: (**a**) PVDF with different crystal structures [68] and (**b**) PVDF with different conformational structures [68]: red, cyan, and blue spheres represent F, C, and H atoms. (**c**) The energy difference between PVDF α and β molecules [69]: the yellow, white, light blue, and gray balls represent gold, hydrogen, fluoride, and carbon atoms, respectively. (**d**) Local annealing treatment and changes in Raman spectra of materials before and after annealing treatment [69], (**e**) mechanical stretching and images of crystal phase changes before and after stretching [70], (**f**) different ways of electrode polarization [71], (**g**) the energy difference between the molecular structure of copolymers and those with different phases and isomorphic anti-plane phases with different proportions of TrFE co doping ΔE. Among them, 3/1-helix is the most stable [72].

According to the sources, we divided polymers into two types: synthetic polymers (SP) and natural polymers (NP). Due to different energy storage modes of SP, we further divided them into three categories: dielectric polymers, conductive polymers and ionic hydrogels. This study aims to expound upon the power generation characteristics exhibited by each type of polymer within different environmental energy generation systems, along with the corresponding strategies for augmenting their performance. In this section, the development of dielectric polymers, which play a crucial role in piezoelectric and triboelectric nanogenerators, is discussed, as shown in Figure 3.

### 3.1. Dielectric Polymers for PENG

**Figure 4:**
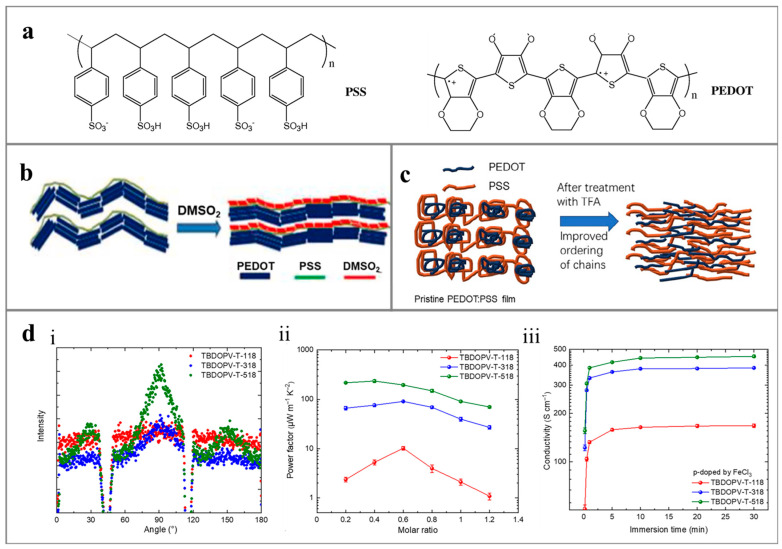
Schematic diagram of PEDOT: PSS structure: (**a**) Schematic diagram of PSS and PEDOT molecular structure [74], (**b**) The structure of PEDOT: PSS before and after treatment with dimethyl sulfone (DMSO_2_) [75], (**c**) Trifluoroacetic acid (TFA) [76] and (**d**) The effect of the degree of π-π contact between chains on conductivity: (i) The microstructure of intrinsic films of different modified polymers was studied using grazing incidence wide-angle X-ray scattering (GIWAXS) technology. The diffraction signal Qr ≈ 1.5 Å^−1^ showed the anisotropic distribution of TBDOPV-T-518 in the layered side chain stacking material, indicating the positive orientation of TBDOPV-T-518 π stacking and the formation of ordered anisotropic arrangements on the molecular side chains. Changes in conductivity of TBDOPV-T-518 when using (ii) N-DMBI and (iii) FeCl_3_ as dopants, respectively [77].

PVDF (polyvinylidene fluoride) is a semi-crystalline polymer with various crystal forms, making it the most used polymer material in piezoelectric nanogenerators due to its excellent dielectric properties and piezoelectric effects [73]. The structure of PVDF is depicted in Figure 4a. The main chain of PVDF contains a strong polar group (–F), allowing the formation of a permanent molecular dipole between the polymer chain and the group. PVDF exhibits five crystal forms, including α-ε, and three conformations, namely TGTG, T_3_GT_3_G and TTTT [68]. Among them, the β-form of TTTT has the largest electric dipole moment due to the symmetrical arrangement of the electronegative fluorine group (–F) and the electropositive alkyl group (–CH_2_) at both sides of the main chain. The molecular dynamics simulations by Chen et al. [69] revealed that α-PVDF is the predominant component in the molten crystal of PVDF due to its lower energy (difference of ~58 meV) compared to β-PVDF. Consequently, in PVDF-based piezoelectric materials, it is essential to maximize the proportion of TTTT conformation and β-PVDF.

The augmentation of the β phase content in polyvinylidene fluoride (PVDF) can be achieved through diverse methodologies, including heat treatment (annealing), stretching, and electric field polarization. Annealing alters the internal molecular structure of the polymer, promoting the transformation from α phase to β phase crystal. For instance, researchers have utilized continuous wave laser irradiation on PVDF films sandwiched between Au nanoparticles (Au NPs) and a gold film to locally heat PVDF, increasing the proportion of the β phase [69]. He et al. [78] facilitated the α to β conversion by integrating Langmuir–Blodgett molding technology with hot pressing, enabling the production of PVDF ultrathin films with a large area, smooth surface, commendable dielectric properties, and piezoelectric characteristics. Another viable method for inducing the transition from α phase to β phase involves mechanical stretching, altering the local mechanical stress on the PVDF molecular skeleton bond [70,79]. As illustrated by Lin et al. [70], the mechanical stretching of α-PVDF film at 80 °C, with a stretching ratio of 1.3, induced a transition in the crystal form from spherical to braided, ultimately yielding a pure β-PVDF film. Complementary to the mechanical stretching approach, the enhancement of the β phase content in fibers can be achieved through diverse spinning techniques, including melt spinning [80], wet spinning [81], and electrospinning [82]. These techniques enable the fabrication of fibers with varied morphological structures, encompassing solid [82], core-shell [83], hollow structures [84].

Furthermore, to enhance the piezoelectric properties, adjusting the structure of piezoelectric materials through copolymerization, doping, and other methods proves effective. Copolymerization, for instance, offers improvements in the piezoelectric properties of PVDF-based dielectric polymers by modifying the polymer chain length and inter-chain distances, thereby influencing the coordination between the crystalline and amorphous phases. Commonly co-doped materials in PVDF-based compounds include trifluoroethylene (TrFE), chlorofluoroethylene (CFE), and fluorinated alkyne (FA) [85]. In a study by Liu et al. [72], they identified a morphological phase boundary (MPB) in P (VDF-TrFE) copolymers, similar to that found in inorganic piezoelectric crystals. By varying the content of VDF in the polymer, they induced a conversion from a 3/1 helical conformation to a TTTT conformation, resulting in the highest piezoelectric coefficient, |d_33_| = 63.5 pC/N. By introducing fluorinated alkynes (FA) into P (VDF-TrFE-CFE), Chen et al. [86,87] achieved the conversion of TG bonds and TT bonds under low electric field, suppressing polarization reorientation between different crystal directions. This approach yielded an impressive piezoelectric coefficient, |d_33_| = 1050 pm/V, marking a significant achievement in PVDF-based dielectric polymer materials.

In addition to copolymerizing doped monomers, the incorporation of solid or liquid nanofillers into PVDF-based composites serves to refine the internal structure of the composites, aiming to enhance the piezoelectric properties of the dielectric polymers. These nanofillers can be categorized based on their primary functions: (1) Inorganic ceramic particles: inorganic materials exhibiting a high piezoelectric effect, such as ZnO, PZT, BTO and BaTiO_3_ [88,89], directly contribute to the enhancement of piezoelectric properties in the composites. (2) Conductive materials: materials like MWCTs, CB, and Ag-NWs facilitate the movement of carriers within the composites, concurrently reducing volume internal resistance. (3) Highly deformable nanodroplets: nanodroplets characterized by high deformability, such as liquid gallium (Ga) [90] and 1H,1H,2H,2H-perfluorodecyltriethoxysilane (PFOES) [91,92], play a crucial role in promoting the internal stress transfer within the composites.

### 3.2. Dielectric Polymers for TriboENG

The triboelectric performance is intricately linked to the piezoelectric and dielectric characteristics of the material. A material with larger piezoelectric coefficient results in significant polarized electric field generation during the compression deformation of the two friction layer materials in contact under inertial force. This phenomenon leads to the movement of electronic energy levels, an increase in energy level difference, and a heightened occurrence of charge transfer [93]. Consequently, the triboelectric properties are predominantly determined by the material’s piezoelectric properties during such conditions [93]. Alternatively, when a material possesses a substantial dielectric coefficient, it exhibits a larger charge storage capacity. In the triboelectric process, this results in a reduced flow of triboelectric charges from the surface of the negative triboelectric material to the positive triboelectric material’s surface. Consequently, triboelectric materials with a higher dielectric coefficient experience a higher surface charge density [94]. In this scenario, the triboelectric properties are primarily influenced by the dielectric properties of the material [93]. Compared to materials with higher dielectric constants or enhanced piezoelectric polarization, materials possessing both moderate piezoelectric and dielectric properties exhibit lower triboelectric characteristics. Therefore, it is essential to choose appropriate dielectric materials within a suitable temperature range. This ensures that the transfer of triboelectric charges is predominantly governed by a single factor, either the polarization field or the dielectric field [93].

The dielectric properties of a material primarily stem from the delocalization of dipoles within the material. When dipoles are arranged randomly and at a considerable distance from each other, the interaction force between them is small, resulting in a large transmission resistance and, consequently, a higher energy storage density [93,95]. Currently, methods to enhance the dielectric properties of materials can be categorized into three groups. This first group brings the interior of the composite closer to percolation, leading to a substantial increase in the dielectric constant, while requiring a small filling amount. However, the strong polarization interface between the composite material and the filler increases the dielectric loss, necessitating the introduction of an insulating coating layer [96] or the design of a nucleation shell structure for the conductive filler [95]. The second group involves the incorporation of high dielectric constant insulating material into low dielectric polymer. By adding a high dielectric constant insulating material to a low dielectric polymer, the dielectric constant of the composite can increase significantly when the filler concentration surpasses a certain threshold (32.6–33.2%) [97]. Nevertheless, the rapid increase in the polarization electric field within the material, due to electrostatic interactions between dipoles, may lead to current leakage. Hence, it is crucial to design the filler structure and the interface between them in the second group [98]. Finally, the third group involves ion implantation to modify dipoles in dielectric polymers. Ion implantation technology offers a method to transform polar non-dielectric materials into dielectric materials by establishing new polar bonds in dielectric polymer molecules. For instance, Fan et al. [99] implanted N ions into PTFE and FEP, resulting in modified PTFE and FEP with a larger dielectric constant and surface charge capacity. This is attributed to the formation of new carbon-nitrogen double/triple bonds, leading to a 7.8-fold and 4.6-fold increase in the number of frictional charge transfers in the corona charging process, respectively.

## 4. Conductive Polymers for Environment-Induced Power Generation

Conductive polymers, characterized by semi-crystalline structures with conjugated π-π bonds, find broad applicability in thermoelectric power generation systems. These materials enable the collection and conversion of heat energy through the movement of carriers (electrons/holes) along the temperature gradient. Given that enhancing the conductivity of conductive polymers has minimal impact on the material’s thermal conductivity, this section focuses on methods to improve the conductivity of conductive polymers, thereby enhancing their thermoelectric properties, as shown in Figure 4.

Enhancing the content of the crystalline phase in conductive polymers represents a strategy to augment the material’s conductivity. The conductive principles differ between the crystalline and amorphous phases of the conductive polymer. Within the crystalline portion, electrical conductivity is achieved through the transport of delocalized electrons in the sp2 structure between polymer chains. Conversely, in the amorphous phase, electrons are transmitted through hopping and tunneling effects, leading to lower material conductivity [100]. Thus, elevating the proportion and continuity of the crystalline phase in conductive polymers can enhance charge transfer performance, thereby improving the thermoelectric properties of the material. For example, the p-type conductive polymer PEDOT, with holes as carriers, exhibits good electrical conductivity. However, PEDOT’s solubility is limited, necessitating the addition of a negatively charged polyelectrolyte PSS to enhance the PEDOT content in water. Introducing organic solvents, ionic liquids, surfactants, salts, and other substances promotes the aqueous solution separation of PEDOT and PSS, leading to improved crystallinity and conductivity of PEDOT [101].

Within the crystalline phase, the enhancement of polymer conductivity primarily focuses on two aspects: increasing the number of mobile carriers in the material and improving the unidirectional transport capacity of carriers. Doping stands out as a method to augment the number of mobile carriers within the material. The effective introduction of P/N-type dopants into the intrinsic conductive polymer promotes the number of carriers in the material, thereby enhancing its conductivity. For instance, Bounioux et al. [102] achieved a maximum conductivity exceeding 1000 S/m by adding a P-type dopant (FeCl_3_) to the composite of poly(3-hexylthiophene) (P3HT) and single-walled carbon nanotubes (SWCNT), without compromising the thermal conductivity of the material. In N-type polymers with electrons as carriers, although the conductivity is generally low, the introduction of appropriate dopants can significantly improve conductivity. For example, Xiao et al. [103] utilized the polymer (PEI-PFA) as the N-type dopant of SWCNT, achieving a conductivity of 1641.22 mS/cm and an electrical power factor of 115.77 mS/cm after treatment with NaOH. However, it is crucial to note that while increasing the charge carrier density and mobility of conjugated polymers enhances the material’s conductivity, it also affects the Seebeck coefficient. Excessive doping levels can reduce the Seebeck coefficient, leading to a decrease in the ZT value of the material [104]. Therefore, optimizing the doping level for the specific material system, referred to as the optimal doping level, is necessary to achieve the best thermoelectric performance [100].

Modifying the polymer chain structure offers a means to enhance the unidirectional transport capacity of carriers, thereby improving the conductivity of polymer molecules and achieving superior thermoelectric properties. Research indicates that an organized interconnected network fosters the unidirectional transport of carriers along the main chain of the conductive polymer, leading to heightened polymer conductivity [105]. By reducing the crystallization rate of the side chain and adjusting the position of the side chain branching point in relation to the main chain, it becomes possible to facilitate the formation of π low side contacts between the main chains, ultimately enhancing the degree of order in charge transfer between chains. Consequently, this approach has yielded high conductivity, reaching up to 100 S/cm (400 S/cm) in n-type and p-type polymers, respectively [77]. In the process of leveraging dopants to enhance conductivity, the distribution of small molecule dopants is typically random, causing distortion of the ordered microstructure in the conductive polymer and impacting the charge transfer process. To address this, dopant molecules can be selectively incorporated into the electrically inactive region of the film occupied by the insulating flexible side chain through solid-state diffusion. This strategic incorporation serves to enhance the transport properties of the conductive polymer.

## 5. Ionic Hydrogels for Environment-Induced Power Generation

Ionic hydrogel materials, characterized by a polymer molecular network as the scaffold and an aqueous solution or intrinsic conductor ionic liquid as the solvent, find versatile applications across various nanogenerator, including piezoelectric, thermoelectric, and humidity power generation [106]. The power generation performance of systems utilizing ionic hydrogel as the power generation material relies on the migration of anions and cations within the material [107]. This section primarily outlines approaches to enhance the effective mobility difference, focusing on material improvement and device structure improvement. Additionally, we provide insights into the practical applications of nanogenerators utilizing various ionic hydrogel materials. While hydrogel materials can be employed in environmental energy nanogenerator systems driven by different forces arising from the distinct mobility of internal anions and cations, it is crucial to acknowledge that hydrogel-based materials manifest diverse power output characteristics in various systems, thereby influencing their applications accordingly.

**Figure 5:**
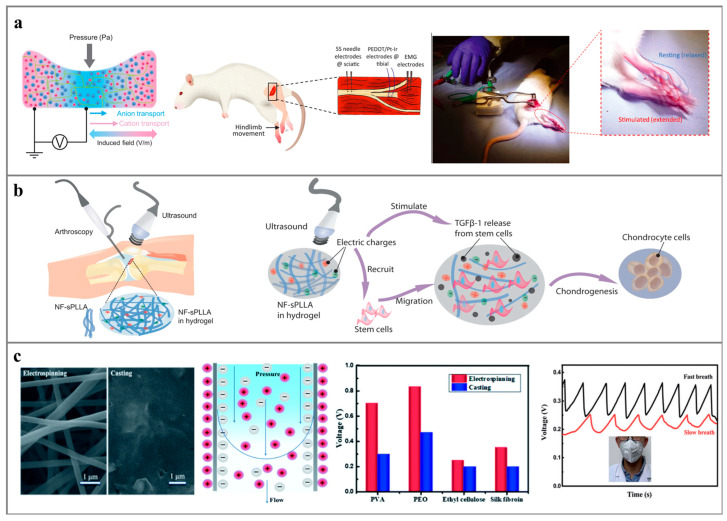
Ionic hydrogels for different environment-induced power generation and their applications: (**a**) demonstration of piezoionic neuromodulation in a rodent modelt [44], (**b**) injectable and biodegradable PLLA-PENG for osteoarthritis treatment [109]. (**c**) PSSA/PAN-MENG made by electrospinning is used as a self-powered respiratory detector [110].

In the PENG system, the gel exhibits a substantial current output (>10 electrical systems) and a modest voltage (<0.1 V) when subjected to external pressure or tension [44]. The power generation performance varies with fluctuating vibration pressure, aligning with the physiological nerve conduction processes within organisms. This characteristic renders it highly promising for applications in ion skin, nerve interface technologies, and implantable medical devices [44], as shown in Figure 5a. For instance, Vinikoor et al. [108] proposed a short nanofiber hydrogel composed of poly-L-lactic acid (PLLA) suitable for direct injection into the body, enabling minimally invasive implantation. The hydrogel, when implanted in bone and joint regions, generates a micro-electric field under compressive conditions, thereby promoting self-regeneration of adjacent damaged tissues (Figure 5b). In thermoelectric power generation systems, hydrogel materials can harness low-grade thermal energy, such as human body temperature, and convert it into electrical energy. These materials are designed to be stretchable and flexible, making them compatible with wearable devices and biosensors for self-powering small appliances [109].

Due to bulk hydrogels typically lacking air permeability and flexibility, they are commonly combined with electrospinning technology, transforming hygroscopic hydrogel materials into micro-nanofibers, significantly enhancing the hydrogel material’s breathability. Taking humidity power generation systems as an example, the integration of hydrogel materials prepared in combination with electrospinning technology serves a dual purpose (Figure 5c). On one hand, it increases the contact area between the hydrogel material and water vapor, facilitating H^+^ dissociation and amplifying ion concentration differences between distinct humidity levels. On the other hand, the cooperative action of ion-selective channels within the fiber fabric can enhance the output performance of moist-electric generators (MENGs) [110]. MENGs can be driven by water vapor in the air and have a wide range of energy sources. Typically, they can be combined with thermoelectric power generation systems by adjusting the direction of the temperature gradient field, serving as a self-powered power supply for wearable devices [111,112].

## 6. Natural Polymers for Environment-Induced Power Generation

Natural polymers (NPs) are a class of natural macromolecules formed by polycondensation reactions using biological small organic molecules such as monosaccharides, amino acids, deoxyribonucleotides, and ribonucleotides as monomers, which can be applied to systems such as PENG, OPNG, and MENG. Depending on the material, NPs in nanopower systems can be divided into two categories: cellulose and protein. According to Chae et al. [113], the piezoelectric coefficients d_33_ of cellulose and protein can reach 210 pC/N and 22 pC/N. However, the natural polymer materials are non-toxic, biocompatible, have excellent degradability, and are more suitable for wearable devices as well as implantable medical devices. Therefore, this section focuses on the application of natural polymer materials in different nanogeneration systems.

**Figure 6:**
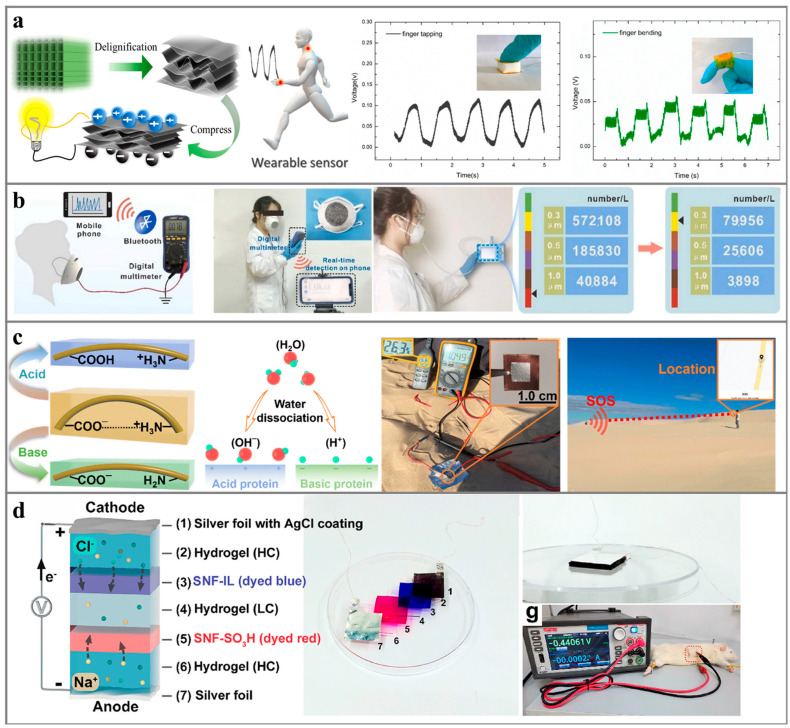
Applications of natural polymer nanogenerators: (**a**) cellulose-based PENG with sponge structure is used as a wearable smart pressure sensor [116], (**b**) the self-powered air filter manufactured by CA/Ni-HITP TriboENG and the comparison of the number of submicron particles inside and outside the mask after wearing it for 2 h [117], (**c**) the structure of the acidic/alkaline whey protein film, the principle of power generation, and the picture of it powering the wireless position tracker at low humidity [118], (**d**) introducing ionic liquids into the internal solvent of the gel [119].

There are electron-rich and electron-poor groups in the molecular structure of NP. The arrangement between the different electrical groups can affect the piezoelectric activity as well as the triboelectric properties of the material. For example, by doping cellulose and other piezoelectric materials, electron-rich groups (hydroxyl, amino, alkoxy, etc.) and electron-poor groups (acyl, aldehyde, etc.) can be arranged side by side on both sides of the polymer chain, making the material dipole-rich. Under the same strain conditions, a larger piezoelectric field is generated, resulting in excellent piezoelectric properties [114,115]. In addition, by chemically modifying the surface of the material and doping polar fillers into the interior of the material, the affinity for electrons and the pore structure of the power generation material can be changed, thus improving the triboelectric properties [115]. For example, Sun et al. [116] chemically treated balsa wood by removing lignin and hemicellulose to get cellulose with a rich pore structure of cellulose. The pore structure increased the compressibility of cellulose-based PENG, making the performance of cellulose-based PENG 85 times better than that of virgin wood. As shown in Figure 6a, the cellulose-based PENG can eventually be applied in wearable smart pressure sensors to monitor human movement. In addition, Fu et al. [117] have improved the performance of cellulose-based PENG by introducing a conductive metal-organic skeleton into the cellulose aerogel to fabricate TriboENG. Ni-HITP with electron rich groups (amino groups) and many porous structures pro-motes electron transfer on the surface of CA/Ni HITP, increases the frictional contact area between frictional materials, and improves the power generation performance of CA/Ni-HITP TriboENG. Moreover, the self-powered air filter manufactured by CA/Ni-HITP TriboENG has an excellent air filtration effect, with removal rates of PM1.0, PM0.5, and PM0.3 are 98.4%, 97.3%, and 95.0%, respectively (Figure 6b).

The molecular structure of proteins contains abundant amino and carboxyl groups, which have excellent hydrophilicity and are widely used in OPNG and MENG. The technology is also expected to power implantable medical devices due to the good biocompatibility and biodegradability of the protein in biological tissues. The directional movement process of ions can be enhanced by modulating the surface charge density and internal nanopore distribution of protein materials [120]. For instance, Zhu et al. [118] obtained acidic/alkaline whey protein membranes with positive/negative surface potentials, respectively, by treating proteins with solutions of different pH, and the higher surface potential promoted the separation of anion and cation after hygroscopic hydrolysis. The MENG using alkaline whey protein membrane achieved an open-circuit voltage of 1.05 V (after surface plasma treatment of the hydrophilic), and it powered the wireless location tracker under extremely dry conditions with a relative humidity of 26% (Figure 6c). Lin et al. [119] treated SNF membranes with ultra-low molecular polylysine and sulfoacetic acid/3-(5-carboxy-propyl)-1-methylimidazolium bromide and obtained SNF-SO_3_H and SNF-IL, which are richly positively and negatively charged, respectively (Figure 6d). The high surface charge density of SNF-SO_3_H and SNF-IL endowed them with the strong ion-selective ability, and the OPNG with SNF-SO_3_H/SNF-IL achieved a power density of 0.59 mW/m^2^ in the low concentration range (10 mM NaCl/0.001 mM NaCl).

## 7. Performance Enhancement Methods of Polymer-Based Nanogenerators

### 7.1. Improvement of Electrical Conductivity and Thermal Conductivity

In previous investigations, thermoelectric materials have been broadly categorized into two groups: inorganic materials and organic materials. The assessment of their performance typically relies on the dimensionless measurement of the thermoelectric figure of merit (ZT) [121,122]. Enhancing the power generation performance of thermoelectric generators hinges on improving the ZT value of thermoelectric materials, according to Equation (8), necessitating materials with high electrical conductivity and low thermal conductivity. Among them, the thermal conductivity satisfies Equation (9) [123]. Inorganic materials commonly exhibit superior thermoelectric properties, yet they lack flexibility and are exceedingly brittle, hindering device assembly and miniaturization [124]. In inorganic conductors, the thermal conductivity induced by electron movement is directly linked to the material’s electrical conductivity of the material [123]. Consequently, improving the material’s ZT value involves strategies such as material doping [46,125] or reducing the material dimensions [126,127]. In organic polymers, the primary influence on thermal conductivity is the result of phonon vibration, and the enhancement of conductivity (*σ*) has little impact on *λ* [128]. Organic thermoelectric (TE) materials, characterized by flexibility and biocompatibility, are advantageous for harnessing human heat.

(8) $$ZT=Z\times T=\frac{S_{p,n}^{2}\sigma_{p,n}T}{\lambda_{p,n}}$$

(9) $$\lambda =\lambda_{el}+\lambda_{ph}+\lambda_{am}$$

where *S* represents the Seebeck coefficient. *σ* represents the conductivity. *λ* represents the thermal conductivity. *p* and *n* represent the carrier types, and *T* is the absolute temperature. The terms $\lambda_{el},\;\lambda_{ph},\;\lambda_{am}$ represent the contribution of electron, phonon, and dipole motion or vibration to thermal conductivity, respectively.

**Figure 7:**
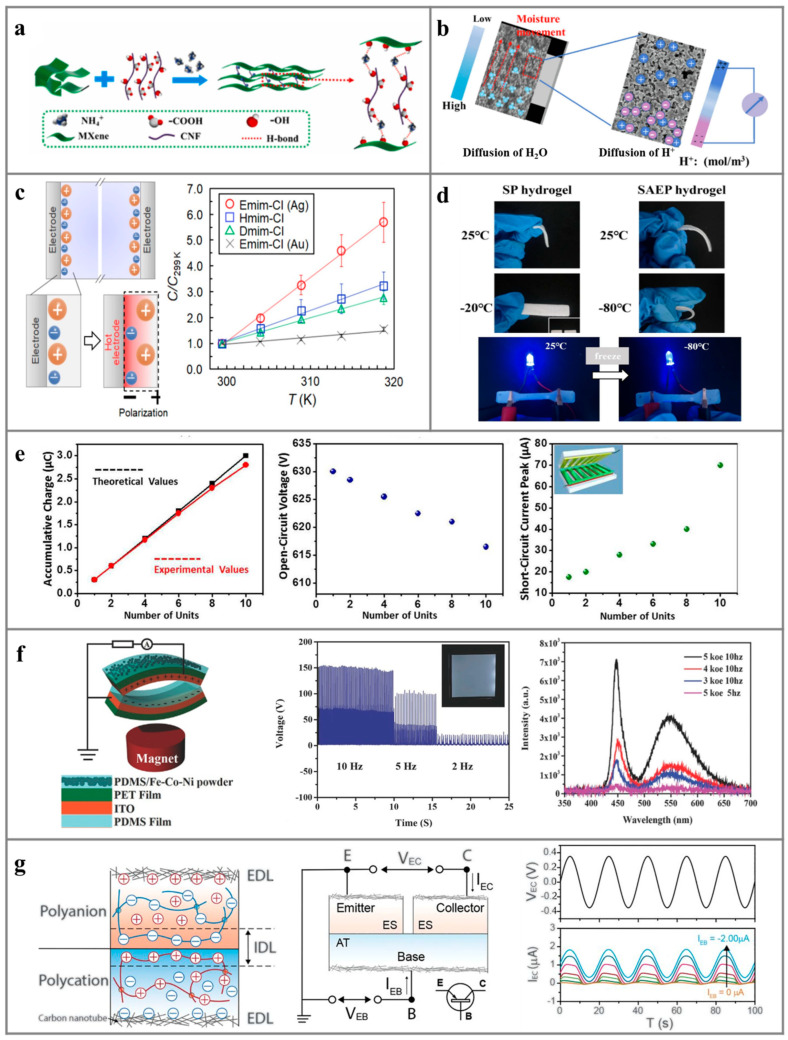
Performance enhancement methods of polymer-based nanogenerators: (**a**) utilizing charged fragments [131] or (**b**) utilizing other substances possessing micro-nano pore structures [33] to creat a single charge channel, (**c**) utilizing ions with smaller radii as solutes [54], (**d**) introducing ionic liquids into the internal solvent of the gel [134], (**e**) configuring with a fence-like structure on the sliding surface of TriboENG [135], (**f**) increasing contact pressure and accelerate friction frequency to obtain a larger effective contact area [136], (**g**) creating an ion rectifier junction akin to a semiconductor [137].

To enhance the electrical conductivity of ionic hydrogel materials and optimize power generation performance, improvements primarily focus on mitigating ion transport resistance and augmenting ion mobility differences. Currently, researchers employ two primary strategies to enhance the disparity in ion mobility: the construction of ion channels and the expansion of ion concentration gradients. The ion channel, by obstructing homoelectric ions and selectively permitting anti-electric ions to pass through, enables the augmentation of the disparity in the mobility between anions and cations. Constructing ion channels can be achieved through two approaches. One method involves creating a single charge channel within the hydrogel skeleton structure utilizing charged fragments [129,130]. For example, Zhao et al. [131] introduced two-dimensional MXene sheets into a polyurethane-ionic liquid composite (Figure 7a). The negatively charged oxygen-containing groups on the MXene surface selectively screen anions of similar charge, resulting in a notable increase in net cation flux. The power generation performance of this material (219 mV) significantly surpasses that of the MXene pressure sensor lacking channels (38 mV). However, it is essential to ensure sufficient spacing between hydrogel chains during the construction of ion transport channels to mitigate the adverse effects of densely bonded networks on ion-conductivity [132]. Another approach involves integrating hydrogel material with other substances possessing micro-nano pore structures [133]. For example, Yan et al. [33], coated a portion of the titanium oxide nanoparticle film with a cobalt hydrogel featuring strong hygroscopicity (Figure 7b). Driven by the ion concentration gradient, this configuration achieved a singular power output of 0.95 V (open circuit) and 0.1 mA (short circuit) due to the overlapping electric double layer effect of ion selection.

### 7.2. Reduction in Ion Transport Resistance

To alleviate ion transport resistance within the gel, a liquid with high boiling point and non-volatility can be used as a solvent to ensure a stable liquid environment [134]. Besides, the solute with the small ion radius within the gel have less transmission resistance (Figure 7c) [54]. Ionic liquids, characterized as molten salt liquids composed entirely of cations and anions with melting points equal to or below 100 °C, serve this purpose [138]. Ionic liquid boasts outstanding electrochemical stability, thermal stability, and low volatility, making it a versatile choice as a solvent or electrolyte [139]. Introducing ionic liquids into the internal solvent of the gel facilitates the maintenance of a stable liquid environment within the gel across a broad temperature range. It enhances the ionic conductivity of the gel in turn. For example, Liu et al. [140] enhanced the antifreeze ability and thermal stability of PVA hydrogels by utilizing a binary liquid of 1-ethyl-3-methylimidazolium acetate (EMImAc) and water as a solvent. This hydrogel exhibited functionality in a wide temperature range (−50–95 °C) and achieved higher conductivity (2.98 S/m) compared to pure ionic liquids (0.33 S/m). Imidazolium-based ionic liquids, when mixed with ethylene glycol/water, further enhanced the solvent’s frost resistance (down to −128.9 °C) and moisture retention (88.10% weight after 20 days) (Figure 7d) [134]. In addition, smaller ions with reduced radii encountered less resistance during movement, enhancing the material’s conductivity (Figure 7c) [54]. For example, Horisk et al. [54] utilized imidazolium cations with ethyl, hexyl, and decyl side chains binding to chloride ions as the electrolyte solution for ionic hydrogels. Experimental findings indicated that the imidazolium cation with an ethyl side chain achieved greater ionic conductivity (1.6 mS/m) due to its lower moving resistance, in contrast to the imidazolium cation with a decyl side chain (1.6 mS/m) (0.63 mS/m).

### 7.3. Utilization of Composite Polymers

In addition to the selection and design of power generation materials, the interface between the power generation material and the electrode plays a crucial role in determining the output performance of the mechanical energy nanogenerator. In the context of triboelectric systems, the sliding surface is usually configured with a fence-like structure. This design enhances the energy harvesting efficiency of the TriboENG during unidirectional movement of the friction layer, facilitating increased charge transfer (Figure 7e) [135,141]. Additionally, an increase in the contact area during friction can facilitate more electron transfer. This occurs when the material, due to a design featuring a hemispherical or pyramid-shaped structure, has a larger specific surface area. Alternatively, under conditions of high contact pressure and fast friction frequency (Figure 7f) [135,141], the material exhibits a greater momentum impulse at the moment of contact. This results in greater surface deformation of the friction layer, leading to a larger effective contact area [142,143] and, consequently, achieving enhanced triboelectric performance.

By augmenting the ion concentration gradient at both extremities of the material, ions experience an increased impetus for movement, thereby enhancing the effective ion transfer rate within the hydrogel. There are two methodologies for establishing the ion concentration gradient: asymmetric humidity environment and asymmetric distribution of oxygen-containing functional groups. The first methodology involves asymmetric humidity conditions on both sides of the power generation material. In the context of the humidity power generation system, ion migration within the hydrogel is predominantly influenced by the ion concentration gradient resulting from disparate moisture levels at opposing ends of the material. Consequently, the humidity gradient can be engineered through physical means such as asymmetric electrode openings or localized incorporation of a moisture-resistant substrate. Alternatively, the gradient distribution of oxygen-containing functional groups can be directly implemented at both ends of the material. Both approaches lead to asymmetric hydrolysis of oxygen-containing functional groups at the material’s extremities, culminating in the formation of an ion concentration gradient [144]. The second methodology involves introducing migrating ions with distinct electrical properties at both termini of the hydrogel. By subjecting the same material to diverse treatments, disparate electrically conductive ions can coexist at the material’s opposing ends, creating an ion rectifier junction akin to a semiconductor at the interface between them (Figure 7g) [137]. In addition, the augmentation of ion gradient can also be achieved by assembling two hydrogels with different electrical carriers [145].

## 8. Conclusions and Future Prospects

Environment-induced nanogenerators are promising of extracting ambient energy in daily life by using soft and easily processable polymers as power-generating medium. This paper provides a comprehensive review of polymers suitable for harvesting ambient energy for power generation, synthetic polymers (dielectric and conductive polymers and ionic hydrogels) with different energy storage modes and natural polymers that are ubiquitous in nature, and reviews the use of these polymers in different types of nanogenerators, including piezoelectric and triboelectric generation using mechanical energy, thermoelectric generation using thermal energy, and osmotic and moist-electric generators using chemical potentials. Although there are several types, the basic principle of polymer-based environmental nanogenerators is that the uneven distributions of environmental physical fields such as pressure, temperature and concentration lead to dipole, electron/hole or ion polarizations inside polymer materials. When electrodes are added to both sides of the polymer medium and external electrical appliances are connected, the potential difference generated by polarization can drive the flow of free electrons, thereby outputting observable voltages and currents.

In this paper, the power generation principles, key materials and performance modulation methods of various polymer-based nanogenerators are reviewed and compared. The key material and main output performance of various nanogenerators are summarized in Table 1. PENGs and TriboENGs using dielectric material have higher voltages than other nanogenerators. However, their performance is closely related to the frequency and intensity of external mechanical motion, and practical applications may be limited. Furthermore, the temperature difference required by ThermoENGs is not high, and it is particularly suitable for utilizing small temperature differences caused by sunlight, indoor and outdoor environmental differences, or human body heat [29]. Thus, ThermoENGs has been gradually extended to practical applications in business and daily life. Moreover, OPNG can use the concentration difference between seawater and fresh water as energy driving force, while MENG can use ambient air humidity. Although the output power density in existing devices is lower, it has the advantages of stable operation and good repeatability, and has been received increasing attentions [33,146].

Although polymer nanogenerators are environmentally friendly, their output performance is still relatively small (~mW/cm^2^) and unstable. The development of hydrogel polymer materials is a promising direction for future nanopower generation [48], such as ionic hydrogels having versatile features such as flexibility, good mechanical properties, high electrical conductivity, and thermal insulation [46], etc. It is also suitable for multi-energy collaborative power generation. Hydrogel matrix composites are also a current research hotspot. For example, composites of polymers with inorganic materials with better dielectric constants or Seebeck coefficients could allow PENGs and ThermoENGs to remain flexible while achieving higher power densities [29,131]. Therefore, we can foresee that with the development of polymer materials, especially hydrogels, the performance of nanogenerators will be significantly improved, and they should expected to achieve commercial applications in fields such as self-powered energy assemblies, portable devices, temperature and humidity sensors, and health monitoring.

## Figures and Tables

**Table 1:** Summary and comparison of output performance of different polymer-based nanogenerators.

Type	Polymer Medium for Power Generation	Materials of Electrodes	V_oc_/V	Isc/μA	Power Density (mW/cm^2^)	Refs
PENG	GaIn NDs/PVDF-TrFE	Al	212	0.04	23.4	[90]
	BTO/PVDF/PFOES	Ni	102	10	0.07	[92]
Trbo ENG	PVDF-TrFE	AgNMW/MWCNTs/PDMS; Al	508	16.5	0.528	[142]
	Ecoflex, Kapton	MXene, PVA	180	1.2	0.033	[147]
	PTFE, PI	Carbon	80	1.7	0.0068	[148]
Thermo ENG	PVA/PEDOT/PAMPS	Au	25.0 mV/K		9.93 mW/(m K^2^)	[23]
	Emim-Cl/PVA	Al	10.1 mV/K			[54]
	SWCNT/PEI-PFA /NaOH	Cu	0.02656 mV/K		0.11577 mW/(m K^2^)	[103]
OPNG	SPEEK	Ag/AgCl	0.349	0.63	0.58	[149]
	Polyelectrolyte hydrogel	Ag/AgCl	0.043	7.5	0.787	[150]
MENG	TiO_2_-Co hydrogel	Carbon	0.95	100	0.00179	[33]
	PVA-PA		0.8	60	0.035	[151]
	CB-LiCl/PVA	Au	0.6	6	0.07 mW/cm^3^	[152]
Thermo ENG and MENG	P(AMPS-SSS_0.5_)	C	1.81 and 126.2 mV/K	<0.5	0.00475 mW/cm^3^ and 15.6 mW/(m K^2^)	[111]
	PSSA-PEDOT: PSS-K_4_Fe(CN)_6_/K_3_Fe(CN)_6_	C	0.94 and 30.0 mV/K	800	7.2 and 0.05 mW/(m K^2^)	[153]
